# Adenosine A_2A_ Receptor Antagonist Improves Cognitive Impairment by Inhibiting Neuroinflammation and Excitatory Neurotoxicity in Chronic Periodontitis Mice

**DOI:** 10.3390/molecules27196267

**Published:** 2022-09-23

**Authors:** Wendan He, Xianlong Xie, Chenxi Li, Huang Ding, Jishi Ye

**Affiliations:** 1 Department of Stomatology, The Affiliated Hospital of Wuhan Traditional Chinese and Western Medicine, Tongji Medical College of HUST, Wuhan 430022, China; 2 Department of General Practice, The Affiliated Hospital of Wuhan Traditional Chinese and Western Medicine, Tongji Medical College of HUST, Wuhan 430022, China; 3 Laboratory for Tumor Genetics and Regenerative Medicine, Department of Oral and Maxillofacial Surgery, The Head and Neurocenter, University Medical Center Hamburg-Eppendorf (UKE), 20246 Hamburg, Germany; 4 Department of Anesthesiology, Renmin Hospital, Wuhan University, Wuhan 430060, China; 5 Department of Pain, Renmin Hospital, Wuhan University, Wuhan 430060, China

**Keywords:** cognition, glutamate, *Porphyromonas gingivalis*, SCH58261

## Abstract

The adenosine A_2A_ receptor antagonist SCH58261 has been reported to have anti-inflammatory effects. However, its role in chronic periodontitis (CP)-induced cognitive impairment, which is associated with *Porphyromonas gingivalis* lipopolysaccharide (*P. gingivalis* LPS), remains unclear. This study investigated the role of SCH58261 in mice with CP-induced cognitive impairment. C57BL/6J mice were used to develop CP model by injecting 0.5 mg/kg *P. gingivalis* LPS into the palatal gingival sulcus of maxillary first molars twice a week for four weeks. The mice were divided into control, *P. gingivalis* LPS (P-LPS), P-LPS + SCH58261, and SCH58261 groups. The passive avoidance test (PAT) and Morris water maze (MWM) were used to assess cognition in mice. Furthermore, CD73/adenosine, neuroinflammation, glutamate transporters, and glutamate were assessed. Compared with the P-LPS group, 0.1 and 0.5 mg/kg SCH58261 increased latency and decreased error times in PAT, but increased platform crossing number in MWM. SCH58261 inhibited microglial activation, and decreased pro-inflammatory cytokines and glutamate levels, but increased GLT-1 and PSD95 expression in the hippocampus. This was the first report of SCH58261 treatment for CP-induced cognitive impairment, which may be related to its anti-inflammatory activities and anti-glutamate excitatory neurotoxicity. This suggests that SCH58261 can be used as a novel agent to treat cognitive impairment.

## 1. Introduction

Chronic periodontitis (CP) is a chronic infectious disease where plaque microorganisms act as the initiating factor. CP has a prevalence rate of >50% in adults [1]. It can cause a systemic inflammatory response and various diseases such as cardiovascular disease, respiratory disease, and central nervous system (CNS) diseases [2,3,4]. However, effective treatments against CP are lacking.

*Porphyromonas gingivalis* is common Gram-negative anaerobic bacteria associated with CP. Lipopolysaccharide (LPS), which is located on the outer membrane of Gram-negative bacteria, is involved in a major pathological pathway of *P. gingivalis* [5]. LPS derived from *P. gingivalis* has been confirmed to induce immune cells to release inflammatory factors, reactive oxygen species, and nitric oxide, resulting in cell death, apoptosis [6,7], and ultimately, cognitive dysfunction [5,8].

Adenosine is an endogenous purine nucleoside that regulates numerous physiological functions by activating the four G protein-coupled receptors A_1_, A_2A_, A_2B_ and A_3_ [9,10]. CD73 (ecto-5-nucleoditase) plays an important role in the immune inflammatory response against various diseases. Specifically, CD73 is the rate-limiting enzyme that involved in the catabolism of adenosine triphosphate (ATP) to produce adenosine [11]. A recent study on experimental autoimmune encephalomyelitis (EAE) suggested that theta-burst stimulation provides neuroprotection in EAE mice by downregulating the CD73 expression [12]. A_2A_ receptor (A_2A_R) is a widely studied receptor associated with CNS diseases owing to its high affinity for adenosine [13]. For instance, Rebola et al. reported that the A_2A_R antagonist SCH58261 prevents LPS-induced neuroinflammation in the hippocampus [14]. Nevertheless, the relationship between CD73 and A_2A_R and its role in CP remains obscure.

Glutamate is associated with learning and memory [15]. Sufficient glutamate levels are necessary to maintain learning and memory; however, excessive glutamate can lead to excitatory neurotoxicity and ultimately cause neuronal damage, resulting in cognitive impairment [16]. The extracellular glutamate level is regulated by reuptake through glutamate transporter-1 (GLT-1) and glutamate and aspartate transporter (GLAST). In addition, TNF-α downregulates the GLT-1 levels via the TNF-α receptor [17]. To the best of our knowledge, the function of glutamate in CP-induced cognitive impairment has not been reported yet.

In the present study, we hypothesized that the overexpression of CD73 leads to increased adenosine production, thus inducing an inflammatory response. Furthermore, the excessive release of inflammatory factor (e.g., TNF-α) leads to decreased glutamate transporter expression, causing an increase in the glutamate level and subsequent cognitive dysfunction.

## 2. Materials and Methods

### 2.1. Animals

Male C57BL/6J (8 weeks old, 20–25 g) were purchased from Vital River Laboratory Animal Technology Co., Ltd. (Beijing, China) and used in the present study. Mice were housed in a temperature (21 ± 1 °C) and humidity (50% ± 5%) controlled room with a 12 h light/dark cycle and free access to food and water. Mice acclimatized to the environment for two weeks before experiment. The experimental protocol was approved by the Animal Ethics Committee of Tongji Medical College of Huazhong University of Science and Technology (ethics approval number: TJH-202003175), and all experiments were performed in accordance with the Guiding Principles for the Care and Use of Animals in Research, the ARRIVE 2.0 guidelines [18].

### 2.2. Chronic Periodontitis (CP) Model and Experimental Protocol

According to previous reports [8], *P. gingivalis* LPS was purchased from InvivoGen company (San Diego, CA, USA, tlrl-pglps) and injected into the palatal gingival sulcus of maxillary first molars twice a week for four weeks at a dose of 0.5 mg/kg to construct CP mice model. The behavioral tests were performed in the light phase.

Experiment Ⅰ: 24 mice were randomly divided into two groups: control group and P-LPS group. The P-LPS group was given 0.5 mg/kg *P. gingivalis* LPS, while the control group was given an equivalent volume of saline. In this case, 28 days after administration of the treatment, cognitive function of the mice after recovery of motor activity were evaluated by open field test (OFT), Morris water maze test (MWM) and passive avoidance test (PAT). After behavioral tests, the mice were sacrificed for molecular biological detection.

Experiment Ⅱ: 48 mice were randomly divided into four groups: P-LPS group, P-LPS + 0.02 mg/kg A_2A_R antagonist SCH58261 (P-LPS + 0.02 SCH) group, P-LPS + 0.1 mg/kg SCH58261 (P-LPS + 0.1 SCH) group and P-LPS + 0.5 mg/kg SCH58261 (P-LPS + 0.5 SCH) group. According to previous reports [19,20], three different doses of SCH58261 (Selleckchem, TX, USA, S8104) were injected intraperitoneally once a day for four weeks. Behavioral tests were same as experiment Ⅰ.

**Figure 1:**
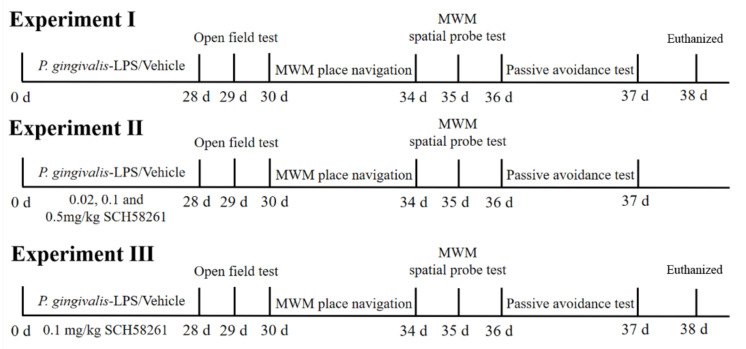
Time-line diagram of study design. Experiment Ⅰ: *P. gingivalis* LPS was injected into the palatal gingival sulcus of maxillary first molars twice a week for four weeks at a dose of 0.5 mg/kg to construct CP mice model. OFT, MWM and PAT were performed on the days 29, 30–35, and 36–37 after *P. gingivalis* LPS administration, respectively. On the day 38, mice were sacrificed for molecular biological detection. Experiment Ⅱ: the CP mice model was established with *P. gingivalis* LPS along with daily intraperitoneal injections of SCH58261 (0.02, 0.1 and 0.5 mg/kg) for four weeks. The behavioral tests were the same as Experiment Ⅰ. Experiment Ⅲ: the CP mice model was established with *P. gingivalis* LPS along with daily intraperitoneal injections of SCH58261 (0.1 mg/kg) for four weeks. The behavioral and molecular biological tests were the same as Experiment Ⅰ. OFT, Open Field Test; MWM, Morris Water Maze; PAT, Passive Avoidance Test.

Experiment Ⅲ: 48 mice were randomly divided into four groups: control group, P-LPS group, P-LPS + 0.1 mg/kg SCH58261 (P-LPS + 0.1 SCH) group and 0.1 mg/kg SCH58261 (0.1 SCH) group. Behavioral tests and molecular biological assay were same as experiment Ⅰ and Ⅱ. The diagram of the experiments was shown in Figure 1.

### 2.3. Behavioral Tests

#### 2.3.1. Open Field Test

The OFT was used to evaluate locomotion and anxiety-like behavior of the rodents [5,10]. The open field chamber was placed in a quiet and dim environment, and mice were placed in the center of chamber and allowed to explore freely for 5 min. There is a camera on top of the chamber to record the motion trail of the mice. The chamber was divided equally into 16 compartments, and the middle 4 compartments were defined as the central zone. Based on the tendency of mice to move along the chamber walls, the time spent in the central compartment was used to reflect the anxiety level of mice. In addition, total distance was recorded to reflect the locomotion of mice.

#### 2.3.2. Morris Water Maze Test

The MWM is performed for hippocampus-dependent tests of spatial navigation and reference memory of rodents [21]. The MWM consists of a cylinder (diameter 1.2 m and height 0.5 m) and the water temperature is maintained at 22 °C. The MWM is divided equally into four parts with different visual cues and one part was named as target quadrant, in which a platform was placed one centimeter below the water surface. In the place navigation test (four trials per day), mice were placed in the maze from one quadrant facing the wall of the pool and allowed to explore for 90 s, and the time to locate the platform was defined as escape latency. Spatial probe test was performed on the day after the place navigation test. After the platform was evacuated, the mice were placed from the quadrant opposite to the target quadrant, and the time spent in the target quadrant and the number of platform crossings were recorded.

#### 2.3.3. Passive Avoidance Test

The PAT was performed to assess passive avoidance learning in mice [22]. The device was divided into light and dark compartments according to the mice’s preference for dark environments. The mice will receive an electric shock (0.6 mA, 2 s) when entering the dark compartment.

Training test was performed on the first day. Mice were placed in the light compartment and after 10 s the partition between the light and dark compartments was opened. The mice were free to explore for 5 min and the time to enter the dark compartment was defined as the latency. A retention test was executed 24 h later. The latency to the dark compartment and the number of electrical shocks (error times) were recorded within 5 min.

The data of the above three behavioral tests were recorded and analyzed by the software (ANY-maze tracking system).

### 2.4. Western Blotting

Radioimmunoprecipitation lysis buffer (Beyotime, Shanghai, China, P0013B) with protease phosphatase mixture were used to lyse hippocampus tissues. Lysates were centrifuged (12,000× *g*, 10 min, 4 °C), and protein quantification of supernatants was performed with BCA kit. Protein samples were separated by 10% SDS-PAGE and transferred onto PVDF membranes, and subsequently blocked with 5% Albumin Bovine V for 1 h at room temperature. The membranes were immunoblotted with primary antibodies overnight at 4 °C: GLAST (1:1000, Santa Cruz Biotechnology, Santa Cruz, CA, USA, sc-515839), GLT-1 (1:1000, Santa Cruz, sc-365634), postsynaptic density (PSD) 95 (1:1000, Santa Cruz, sc-32290) and β-actin (1:1000, Santa Cruz, sc-47778). The membranes were washed with TBST for three times and incubated with HRP-conjugated secondary antibody for 1 h at room temperature. The strips were detected by Molecular Imager VersaDoc MP 5000 System and analyzed with ImageJ software (Version 1.50i, National Institutes of Health, Bethesda, MD, USA).

### 2.5. Enzyme-Linked Immunosorbent Assay (ELISA)

The levels of CD73 (Abbexa, Cambridge, UK, abx117254), TNF-α (Boster Biological Technology, Wuhan, China, EK0527), IL-1β (Boster, EK039) and IL-6 (Boster, EK0411) in the hippocampus were quantified by ELISA kit in accordance with the manufacturer’s instructions.

### 2.6. Measurement of Adenosine and Glutamate Concentration

Hippocampus tissue was homogenized in PBS and centrifuged at 10,000× *g* for 10 min at 4 °C. Next, the supernatants were collected and assayed without dilution via the Adenosine Assay Kit (Cell Biolabs, Inc., San Diego, CA, USA, MET-5090) according to the manufacturer’s instructions. Glutamate was analyzed by a colorimetric enzyme method using a kit (Sigma Aldrich, St. Louis, MO, USA, MAK004), following the company’s instruction sheet, and presented as μmol/mL.

### 2.7. Quantitative Real-Time PCR (qRT-PCR)

The hippocampus was homogenized, and RNA was isolated according to the Total RNA Kit (Beyotime, R0032). Total RNA concentration and purity were detected by a NanoDrop spectrophotometer. RNA was reverse transcribed into cDNA using the cDNA Synthesis Kit (Beyotime, D7170M). The sequences of these primers were as follows: PSD95: 5′-GACAACCAAGAAATACCGCT-3′ (forward) and 5′-GCTTCTAGGG TGTCCGTGTT-3′ (reverse); GLT-1, 5′-CCAAGCTTGGATCACTGCCCTGG-3′ (forward) and 5′-CCAGCCCCAAAAGAGTCACCCACAA-3′ (reverse); β-actin, 5′-AACGCAGCTCAGTAACAGTCC-3′ (forward) and 5′-GTACCACCATGTACCC AGGC-3′ (reverse). RT-PCR analyses were performed using the SYBR Green qPCR Mix (Beyotime, D7260) according to the manufacturer’s protocol: 95 °C for 3 min followed by 40 cycles of 95 °C for 5 s and 60 °C for 30 s. β-actin was used as an internal control, and relative gene expression was calculated using the 2^−ΔΔCt^ method.

### 2.8. Immunofluorescence

Under deep isoflurane anesthesia, the hearts of mice were exposed by dissecting the chest cavity. PBS was used for perfusion to remove blood from the circulation, followed by 4% formaldehyde. After the skull was removed, the brain was placed in 4% formaldehyde for 24 h at 4 °C, followed by 30% sucrose dehydration for 48 h. Cryostat microtome was used to coronally cut the brain into 30 μm section. The sections were washed three times with PBS, and transferred to blocking buffer for 1.5 h at 37 °C. Next, the sections were incubated with rabbit anti-Iba1 (1:200, Abcam, Cambridge, UK, ab178847) and PSD95 (1:100, Santa Cruz, sc-32290) overnight at 4 °C. After washing in PBS for three times, the sections were incubated with Cy3 labeled goat-anti-rabbit and goat-anti-mouse secondary antibodies for 1.5 h at 37 °C, followed by DAPI staining for 10 min at room temperature. Washing the sections with PBS three times and mounted in 70% glycerol. Virtual microscopy slide-scanning system was used to capture images, and the images were cropped and analyzed by ImageJ software (Version 1.50i, National Institutes of Health).

### 2.9. Statistical Analysis

GraphPad Prism 7 (GraphPad, New York, NY, USA) was used for statistical analyses. Quantitative data are expressed as the mean ± standard error of the mean (SEM). The normality of the data was analyzed by Shapiro-Wilk test, and the analysis showed that the data were normally distributed. Differences between two groups were assessed using an unpaired two-tailed Student’s *t*-test. Analysis of variance (ANOVA) was used to test differences between three or more groups, followed by Bonferroni’s post hoc test. *p*-value less than 0.05 was considered statistically significant.

## 3. Results

### 3.1. Effects of P. gingivalis LPS on Mouse Behavior

**Figure 2:**
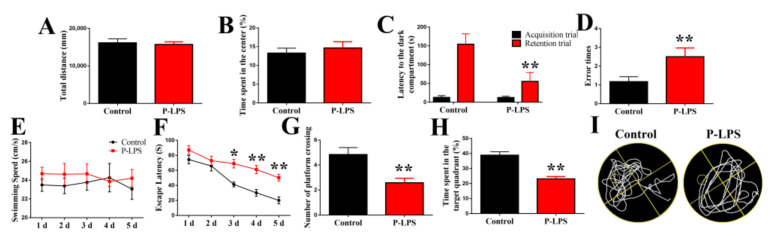
Effects of *P. gingivalis* LPS on the behaviors of mice. Total distance (**A**) and time spent in the center (**B**) were recorded in the OFT on the day 29 after *P. gingivalis* LPS. Latency to the dark compartment (**C**) and error times (**D**) were recorded on the days 36–37 after *P. gingivalis* LPS. Swimming speed (**E**), escape latency (**F**), number of platform crossing (**G**) and time spent in the target quadrant (**H**) were recorded in the MWM test. (**I**) MWM typological trajectories. All data are expressed as the mean ± SEM (*n* = 12 per group). * *p* < 0.05 and ** *p* < 0.01 vs. the control group.

As shown in Figure 2, there were no significant differences in the total distance (Figure 2A) and time spent in the center (Figure 2B) between the control and P-LPS groups. These results indicated that *P. gingivalis* LPS did not affect the locomotion and anxiety levels in mice.

In the training phase of PAT, there was no difference in the latency to dark compartment (Figure 2C) between the control and P-LPS groups, which indicated no difference between these two groups before *P. gingivalis* LPS treatment. The associated memory was retained despite electric shock. As observed in the retention phase of PAT, the P-LPS group showed a decreased latency to the dark compartment compared with the control group (Figure 2C). In addition, the P-LPS group showed more error times in the dark compartment than the control group (Figure 2D). These results demonstrated that *P. gingivalis* LPS damages the passive avoidance learning in mice.

During the place navigation test of MWM, the mice were trained for 5 consecutive days, four trials per day. No significant difference was noted in the swimming speed between the control and P-LPS groups (Figure 2E). Compared with control group, the escape latency significantly increased in the P-LPS group from days 3 to 5 (Figure 2F). During the spatial probe test phase, the platform crossing number (Figure 2G) and time spent in the target quadrant (Figure 2H) significantly decreased in the P-LPS group compared with the control group. Taken together, these data indicate that *P. gingivalis* LPS negatively affects memory in mice. Figure 2I is the movement route of mice in probe trial.

### 3.2. Effects of P. gingivalis LPS on the Adenosine, Inflammatory Factor, Glutamate Transporter, and PSD95 Levels

**Figure 3:**
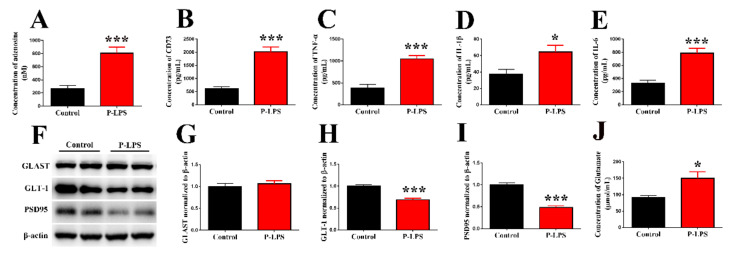
Effects of *P. gingivalis* LPS on the levels of inflammation factors, glutamate transporters and PSD95. The concentration of adenosine (**A**) was determined by fluorescence method. CD73 (**B**), TNF-α (**C**), IL-1β (**D**) and IL-6 (**E**) in the hippocampus of mice were detected by ELISA. The levels of GLAST (**F**,**G**), GLT-1 (**F**,**H**) and PSD95 (**F**,**I**) in the hippocampus of mice were detected by Western blot. The concentration of glutamate (**J**) was detected by colorimetric enzyme method. All data are expressed as the mean ± SEM (*n* = 6 per group). * *p* < 0.05 and *** *p* < 0.001 vs. the control group.

As shown in Figure 3, *P. gingivalis* LPS increased the levels of adenosine, CD73, TNF-α, IL-1β, and IL-6 (Figure 3A–E) in the P-LPS group compared with the control group. These data demonstrate that *P. gingivalis* LPS-induced neuroinflammation maybe associated with increases in the levels of adenosine and CD73, which participate in inflammation and neurodegeneration [23]. According to a previous study, GLT-1 is downregulated by TNF-α [24]. Therefore, we detected the proteins levels of GLT-1 and found that *P. gingivalis* LPS decreases the GLT-1 level (Figure 3F,H) and increases glutamate level (Figure 3J) in the hippocampus of the P-LPS group compared with the control group. In addition, *P. gingivalis* LPS significantly decreased the levels of PSD95 (Figure 3F,I), which may be associated with neuronal damage caused by the increased glutamate level. However, there was no difference in the levels of GLAST (Figure 3F,G) between the control and P-LPS group. These results indicate that the increased level of glutamate, induced by *P. gingivalis* LPS may be associated with decreased GLT-1 but not GLAST.

### 3.3. A_2A_R Antagonist Alleviates P. gingivalis LPS-Induced Cognitive Impairment in Mice

**Figure 4:**
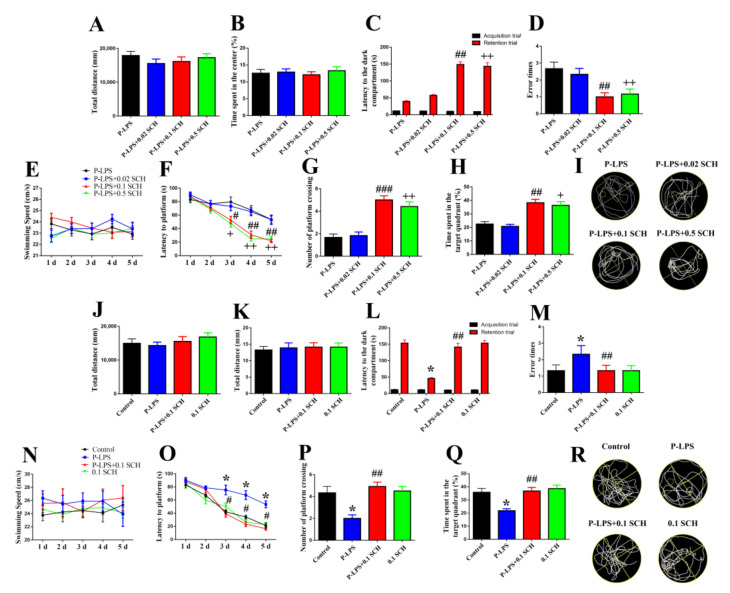
A_2A_R antagonist alleviates *P. gingivalis* LPS-induced learning and memory impaired in mice. Total distance (**A**,**J**) and time spent in the center (**B**,**K**) were recorded in the OFT on the day 29 after *P. gingivalis* LPS. Latency to the dark compartment (**C**,**L**) and error times (**D**,**M**) were recorded on the days 36–37 after *P. gingivalis* LPS. Swimming speed (**E**,**N**), escape latency (**F**,**O**), number of platform crossing (**G**,**P**) and time spent in the target quadrant (**H**,**Q**) were recorded in the MWM test. (**I**,**R**) MWM typological trajectories. All data are expressed as the mean ± SEM (*n* = 12 per group). * *p* < 0.05 vs. the control group; ^#^ *p* < 0.05, ^##^ *p* < 0.01, ^###^ *p* < 0.001, ^+^ *p* < 0.05 and ^++^ *p* < 0.01 vs. the P-LPS group.

In the OFT, there were no differences in the total distance (Figure 4A) and time spent in the center (Figure 4B) among the four groups. This demonstrates that SCH58261 (0.02, 0.1 and 0.5 mg/kg) did not affect the locomotion and anxiety levels in mice.

As shown in Figure 4, no difference was observed in the latency to the dark compartment (Figure 4C) between the four groups during the training phase of PAT. In the retention phase of PAT, SCH58261 (0.1 and 0.5 mg/kg) significantly increased latency to the dark compartment (Figure 4C) and decreased the error times (Figure 4D) compared with the P-LPS group.

There was no significant difference in the swimming speed between the four groups (Figure 4E). In the place navigation test of MWM, SCH58261 (0.1 and 0.5 mg/kg) decreased the escape latency compared with the P-LPS group from days 3 to 5 (Figure 4F). In the spatial probe test phase, SCH58261 (0.1 and 0.5 mg/kg) significantly increased the number of platform crossings (Figure 4G) and time spent in the target quadrant (Figure 4H) compared with the P-LPS group. These results demonstrate that SCH58261 (0.1 and 0.5 mg/kg) alleviates spatial memory impairment-induced by *P. gingivalis* LPS. Therefore, 0.1 mg/kg SCH58261 was the selected for the subsequent behavioral tests and molecular biological assay.

To investigate the effect of 0.1 mg/kg SCH58261 on the behaviors of control mice, we set up groups control, P-LPS, P-LPS + 0.1 mg/kg SCH58261 and 0.1 mg/kg SCH58261 for behavioral tests. Ultimately, we found no significant effect of 0.1 mg/kg SCH58261 on the behaviors of the control mice (Figure 4J–R).

### 3.4. A_2A_R Antagonist Alleviates P. gingivalis LPS-Induced Inflammation in the Mouse Hippocampus

**Figure 5:**
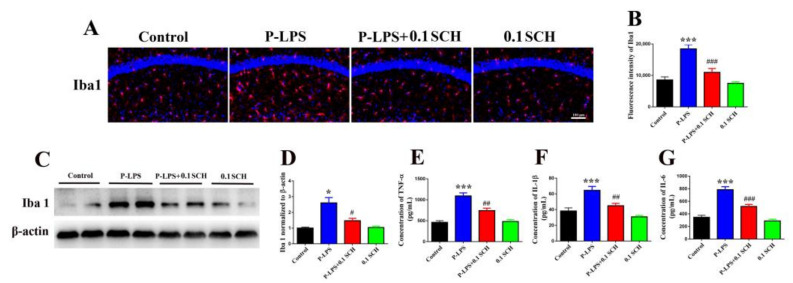
A_2A_R antagonist alleviates *P. gingivalis* LPS-induced inflammation in the hippocampus of mice. The level of Iba1 was detected by immunofluorescence (**A**,**B**) and Western blot, respectively (**C**,**D**). The concentration of TNF-α (**E**), IL-1β (**F**) and IL-6 (**G**) in the hippocampus of mice were detected by ELISA. All data are expressed as the mean ± SEM (*n* = 6 per group). * *p* < 0.05 and *** *p* < 0.001 vs. the control group; ^#^ *p* < 0.05, ^##^ *p* < 0.01 and ^###^ *p* < 0.001 vs. the P-LPS group.

The microglia marker Iba1 was used to reflect activation of CNS inflammation [25]. In this study, *P. gingivalis* LPS significantly increased the density of Iba1 in the hippocampus, whereas SCH58261 decreased it (Figure 5A–D). Furthermore, SCH58261 decreased the levels of TNF-α, IL-1β and IL-6 (Figure 5E–G) in the hippocampus of mice in the P-LPS + 0.1 SCH group compared with the P-LPS group. These data suggest that SCH58261 inhibits neuroinflammation in the hippocampus of mice.

### 3.5. A_2A_R Antagonist Alleviates P. gingivalis LPS-Induced GLT-1 and PSD95 Declines in the Mouse Hippocampus

**Figure 6:**
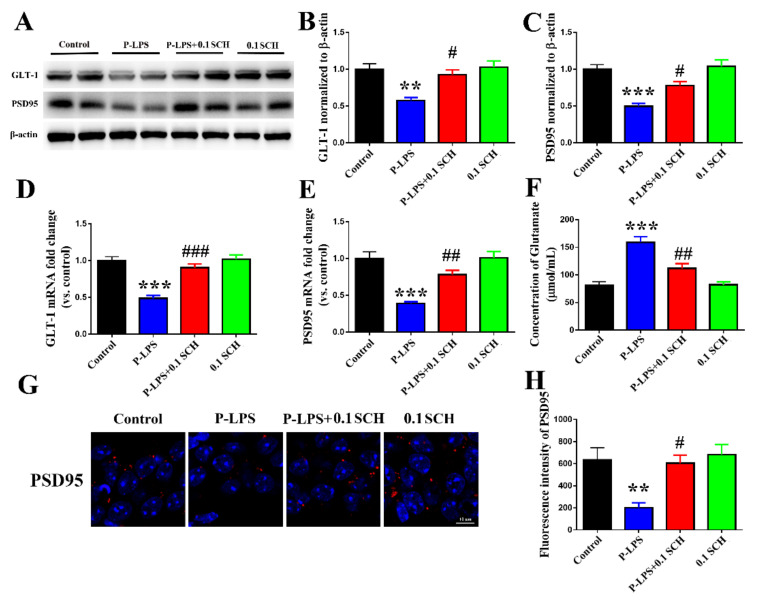
A_2A_R antagonist alleviates *P. gingivalis* LPS-induced GLT-1 and PSD95 decline in the hippocampus of mice. The protein levels of GLT-1 (**A**,**B**) and PSD95 (**A**,**C**,**G**,**H**) in the hippocampus of mice were detected by Western blot and immunofluorescence, while the mRNA of GLT-1 (**D**) and PSD95 (**E**) were detected by qRT-PCR. The concentration of glutamate (**F**) was detected by colorimetric enzyme method. All data are expressed as the mean ± SEM (*n* = 6 per group). ** *p* < 0.01 and *** *p* < 0.001 vs. the control group; ^#^ *p* < 0.05, ^##^ *p* < 0.01 and ^###^ *p* < 0.001 vs. the P-LPS group.

As shown in Figure 6, SCH58261 significantly restored the *P. gingivalis* LPS-induced decreases in the GLT-1 protein (Figure 6A,B) and mRNA (Figure 6D) levels. Moreover, SCH58261 decreased the glutamate level (Figure 6F) and increased the protein (Figure 6A,C,G,H) and mRNA (Figure 6E) levels of PSD95 in the P-LPS + 0.1 SCH group compared with the P-LPS group. Excess glutamate leads to increased Ca^2+^ influx in the neurons by activating *N*-methyl-*D*-aspartic acid receptor (NMDAR), resulting in calcium overload and neuron injury induction [26]. Therefore, we speculated that SCH58261 upregulates the levels of PSD95 by decreasing the glutamate level.

## 4. Discussion

The present study assessed the neuroprotective capabilities of A_2A_R antagonist SCH58261 in CP-induced cognitive impairment. A CP mice model was developed by injecting *P. gingivalis* LPS into the palatal gingival sulcus of maxillary first molars according to a previous study [8]. As evident from the OFT, MWM and PAT results, *P. gingivalis* LPS successfully induced cognitive impairment but not anxiety behaviors in mice. In addition, we found increased levels of adenosine, CD73, inflammatory factors and glutamate and decreased levels of GLT-1 and PSD95 in the CP mice with cognitive impairment. The intraperitoneal injection of SCH58261 decreased the levels of inflammatory factors and glutamate and increased the levels of GLT-1 and PSD95 in the hippocampus of CP mice, ultimately alleviating cognitive impairment. To the best of our knowledge, this study is the first to report the neuroprotective effects of the A_2A_R antagonist SCH58261 mediated by decreasing the glutamate level in the hippocampus of CP mice with cognitive impairment.

The *P. gingivalis* mainly resides in periodontitis plaque, which acts as a reservoir of toxic substances that became transmitted throughout the body [27]. *P. gingivalis* LPS, a highly toxic substances of *P. gingivalis*, reportedly induces cognitive impairment after and intraperitoneal [5] or local injection [8]. In the present study, *P. gingivali* LPS was injected into palatal gingival sulcus of maxillary first molars to develop a mouse model of CP. *P. gingivali* LPS induced cognitive impairment in mice but did not affect locomotion and anxiety behaviors. This observation is consistent with those of the abovementioned studies.

CP is accompanied by systemic inflammation and CNS inflammatory response [28]. Therefore, alleviating the inflammatory response is an effective approach in the treatment of CP. Adenosine is present in all mammals and is mainly released by the nerve endings and glial cells [29]; it can be converted to ATP through the action of CD73 [13]. The levels of adenosine are generally low in physiological conditions, but can increase considerably with CNS damage (e.g., cerebral ischemia and hypoxia) [30]. A recent study has suggested that the CD73 level increases in Parkinson’s disease (PD) models, and limiting CD73-derived adenosine prevents neuroinflammation and improves motor behaviors in PD models [23]. In the present study, the levels of adenosine, CD73 and inflammatory factors increased in the hippocampus of CP mice. Therefore, increased expression of inflammatory factors may be associated with an increased level of CD73 level. Adenosine/A_2A_R signaling reportedly regulates inflammation [14,31]; hence, we used the A_2A_R antagonist SCH58261 to verify the relationship between CD73 and neuroinflammation. We found that SCH58261 inhibited microglial activation and decreased the levels of TNF-α, IL-1β and IL-6, ultimately alleviating cognitive impairment in CP mice. These results indicate that an increased level of CD73 promotes neuroinflammatory response by activating A_2A_R. Moreover, other studies have shown that A_2A_R inactivation promotes microglial inflammatory responses and accelerates disease progression in EAE [32]. Therefore, adenosine/A_2A_R regulates CNS inflammatory response in a context-dependent manner [33].

Glutamate, the most abundant CNS excitatory neurotransmitter, is involved in various physiological regulatory pathways, including neuronal regeneration, synaptic plasticity formation and apoptosis [34]. Although glutamate is an important neurotransmitter for maintaining learning and memory, excessive glutamate levels may induce neuronal damage, known as excitatory neurotoxicity [15]. The levels of glutamate in plasma and cerebrospinal fluid were significantly higher in depressed patients and correlated with the severity of depressive symptoms [35,36]. Clinical evidences demonstrated that ketamine (a potent NMDA receptor antagonist) and esketamine may be effective in treating depression [37]. GLT-1 expression was reduced in the hippocampus of depressed mice, and ketamine improved depression-related symptoms by upregulating GLT-1 expression [38]. Extracellular glutamate level is dependent on its uptake by glutamate transporters (GLT-1 and GLAST) [39]. According to previous study, TNF-α suppresses the expression of glutamate transporters by regulating NF-κB [24]. In the present study, we found an increased glutamate level, decreased expression of GLT-1, but not GLAST, in the hippocampus of CP mice. Conversely, the GLAST, rather than GLT-1, level was reduced in the hippocampus of mice with postoperative cognitive dysfunction [40]. This variability in results might have resulted from different disease models and warrants further relevant studies. SCH58261 restored the decrease in GLT-1 level and subsequently decreased the glutamate level, which indicated that SCH58261 alleviates cognitive impairment in CP mice by relieving excitatory neurotoxicity. TNF-α induces astrocytes to A1-type (neurotoxicity) polarization [41], which impairs the number of synapses and ultimately leads to cognitive impairment [25]. Therefore, we cannot exclude the direct protective effect of SCH58261 on cognitive function, which is mediated through the inhibition of inflammatory responses.

PSD95 cross-talks with NMDAR and its related protein molecules in the signaling pathway, forming a receptor-signaling molecule-regulatory molecule-target molecule complex. This complex can participate in the formation and maintenance of synaptic junctions through the interaction of pre- and postsynaptic adhesion molecules. Furthermore, the complex plays a key role in mediating and integrating NMDAR signaling [42]. Glutamate accumulation leads to excessive activation of NMDAR, resulting in a sustained increase in the intracellular Ca^2+^ concentration, leading to Ca^2+^ overload resulting in neuronal apoptosis and even death [43]. Thus, we speculated that decreased PSD95 expression is related with glutamate accumulation-induced neuronal death and TNF-α decreased the expression of PSD95 by inducing astrocytes to A1-type as mentioned earlier [25].

Current limitations of this study are as follows. First, the use of CD73 knockout mice enabled more rigorous confirmation of the role of CD73. Moreover, we could not rule out the role of other adenosine receptors as only A_2A_R antagonist SCH58261 was used. Second, this study specifically explored the role of the hippocampus, but no other brain regions (e.g., prefrontal cortex) that may play an important role in cognitive impairment [44].

## 5. Conclusions

In the present study, A_2A_R antagonist SCH58261 exerted anti-inflammatory and anti-glutamate excitatory neurotoxicity by blocking CD73/adenosine/A_2A_R pathway, ultimately improving the cognitive impairment caused by CP. This study imply that A_2A_R antagonist may has therapeutic potential for the treatment of cognitive impairment, especially cognitive impairment induced by neuroinflammation and excessive glutamate.

## Data Availability

Not applicable.
